# Human Norovirus Epitope D Plasticity Allows Escape from Antibody Immunity without Loss of Capacity for Binding Cellular Ligands

**DOI:** 10.1128/JVI.01813-18

**Published:** 2019-01-04

**Authors:** Lisa C. Lindesmith, Paul D. Brewer-Jensen, Michael L. Mallory, Boyd Yount, Matthew H. Collins, Kari Debbink, Rachel L. Graham, Ralph S. Baric

**Affiliations:** a Department of Epidemiology, University of North Carolina, Chapel Hill, North Carolina, USA; b Hope Clinic of the Emory Vaccine Center, Division of Infectious Diseases, Department of Medicine, School of Medicine, Emory University, Decatur, Georgia, USA; c Department of Natural Sciences, Bowie State University, Bowie, Maryland, USA; Instituto de Biotecnologia/UNAM

**Keywords:** antibody neutralization, antigenic drift, blockade antibody, immunocompromised, norovirus, receptor binding, viral evolution

## Abstract

Emergent strains of human norovirus seed pandemic waves of disease. These new strains have altered ligand binding and antigenicity characteristics. Study of viral variants isolated from immunosuppressed patients with long-term norovirus infection indicates that initial virus *in vivo* evolution occurs at the same antigenic sites as in pandemic strains. Here, cellular ligand binding and antigenicity of two cocirculating strains isolated from a patient with long-term norovirus infection were characterized. The isolated GII.4 viruses differed from previous strains and from each other at known blockade antibody epitopes. One strain had a unique sequence in epitope D, including loss of an insertion at residue 394, corresponding to a decreased relative affinity for carbohydrate ligands. Replacement of 394 with alanine or restoration of the contemporary strain epitope D consensus sequence STT improved ligand binding relative affinity. However, monoclonal antibody blockade of binding potency was only gained for the consensus sequence, not by the alanine insertion. In-depth study of unique changes in epitope D indicated that ligand binding, but not antibody blockade of ligand binding, is maintained despite sequence diversity, allowing escape from blockade antibodies without loss of capacity for binding cellular ligands.

**IMPORTANCE** Human norovirus causes ∼20% of all acute gastroenteritis and ∼200,000 deaths per year, primarily in young children. Most epidemic and all pandemic waves of disease over the past 30 years have been caused by type GII.4 human norovirus strains. The capsid sequence of GII.4 strains is changing over time, resulting in viruses with altered ligand and antibody binding characteristics. The carbohydrate binding pocket of these strains does not vary over time. Here, utilizing unique viral sequences, we study how residues in GII.4 epitope D balance the dual roles of variable antibody binding site and cellular ligand binding stabilization domain, demonstrating that amino acid changes in epitope D can result in loss of antibody binding without ablating ligand binding. This flexibility in epitope D likely contributes to GII.4 strain persistence by both allowing escape from antibody-mediated herd immunity and maintenance of cellular ligand binding and infectivity.

## Abstract (precis)

Human norovirus causes ∼20% of all acute gastroenteritis and ∼200,000 deaths per year, primarily in young children. Most epidemic and all pandemic waves of disease over the past 30 years have been caused by type GII.4 human norovirus strains. The capsid sequence of GII.4 strains is changing over time, resulting in viruses with altered ligand and antibody binding characteristics. The carbohydrate binding pocket of these strains does not vary over time. Here, utilizing unique viral sequences, we study how residues in GII.4 epitope D balance the dual roles of variable antibody binding site and cellular ligand binding stabilization domain, demonstrating that amino acid changes in epitope D can result in loss of antibody binding without ablating ligand binding. This flexibility in epitope D likely contributes to GII.4 strain persistence by both allowing escape from antibody-mediated herd immunity and maintenance of cellular ligand binding and infectivity.

## INTRODUCTION

Human norovirus infection is a major global health problem, causing over 200,000 deaths annually and accounting for approximately 18 million disability-adjusted life years and 20% of all acute gastroenteritis (AGE) (1, 2). In the developing world, AGE leading to dehydration and malnutrition primarily affects young children (http://www.who.int/news-room/fact-sheets/detail/diarrhoeal-disease). The overall incidence of AGE has been markedly reduced, but norovirus has emerged as the leading cause of AGE in children following the institution of rotavirus vaccination programs (3, 4). Immunocompromised hosts (ICH) represent a uniquely susceptible group that may experience severe and potentially life-threatening norovirus infection with high viral loads or be chronically infected by norovirus and shed virus in the stool for months or even years (5–8). Interestingly, in some individuals there appears to be greater genetic diversity in norovirus isolates from ICH versus healthy adults (5); however, the significance of this finding related to disease burden, transmission, or contribution to the prevalent circulating strains remains unclear.

Belonging to the *Caliciviridae* family, norovirus has a single-stranded, positive-sense RNA genome, with an error-prone RNA polymerase which facilitates antigenic drift. Categorized into genogroups, these viruses contain multiple genotypes, with GII.4 being the predominant cause of human norovirus infections in recent years. The epidemiology of norovirus is characterized by epochal evolution, with genetic variation being concentrated in protruding regions of the major capsid protein consistent with sequential escape from population level immune pressure (9, 10).

Since the mid-1990s, five waves of emergent GII.4 strains have caused pandemic levels of disease. Each successive strain varied from previous strains in neutralizing antibody epitopes A, D, and E, as measured by a surrogate neutralization assay based on antibody blockade of cellular ligand binding. Epitope A is immunodominant and hypervariable, with new strain emergence correlating with evolution in epitope A. Epitope D both mediates neutralizing antibody and ligand binding affinity of histo-blood group antigens (HBGAs), which are cellular cofactors for human norovirus infection. HBGAs are a diverse family of glycosylated macromolecules synthesized by sequential addition of carbohydrate moieties onto a protein or lipid backbone. Host genetics mediate expression of glycotransferases and subsequently types of HBGA expression. Interaction between the α-1,2-fucose of HBGAs, the base molecule for all HBGAs in secretor-positive individuals (11), and GII.4 strains occurs within a conserved carbohydrate binding pocket, the primary ligand binding site (12, 13). Targeted mutagenesis and crystallographic analyses illustrate how residues within epitope D form stabilizing bonds with additional HBGA moieties without modifying the conserved core HBGA binding site (13, 14). In addition to the base α-1,2-fucose (H antigen), GII.4 noroviruses also bind to A antigen, B antigen, and α1,3/4-fucose (Lewis antigen) in strain-specific patterns. Variation in epitope D between GII.4 strains selects for variation in carbohydrate binding affinity and potentially enables expansion of susceptible populations. For example, substitution of D393G of epitope D gains B HBGA binding, expanding the repertoire of cellular ligands (9).

Here, we characterize two GII.4 strains isolated from one stool sample collected from a patient with chronic human norovirus infection. Each strain is antigenically unique and unlikely to be neutralized by herd immunity to the most recent GII.4 pandemic strains. In-depth study of the capsid identifies novel mutations in epitope D and expands understanding of how sequence variation within epitope D can impact antibody neutralization and ligand binding, factors that drive viral infectivity.

## RESULTS

**FIG 1:**
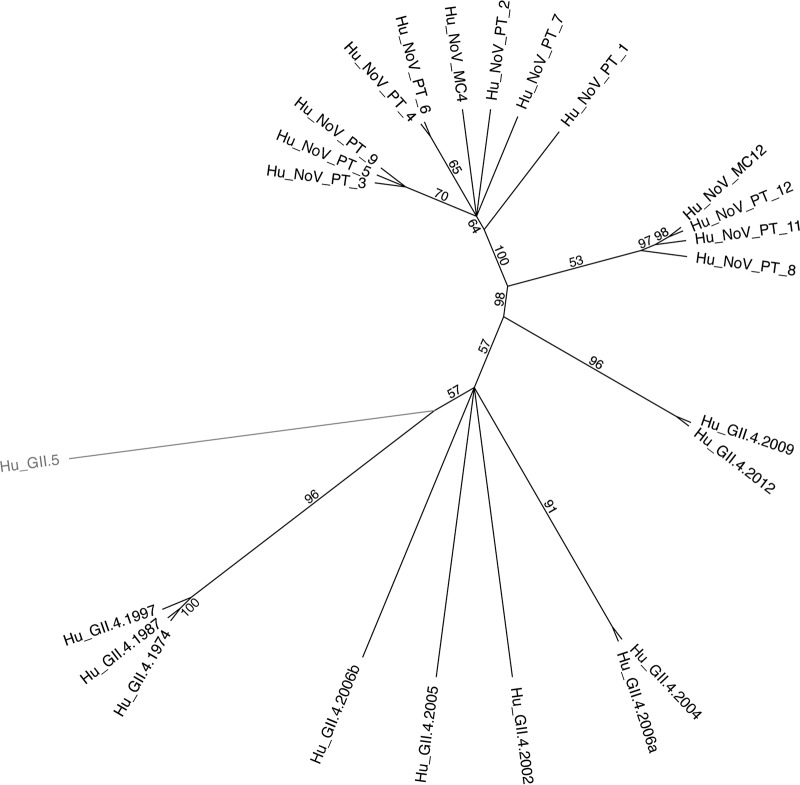
Human norovirus GII.4 phylogenetic tree. A radial phylogram for norovirus GII isolates, including strains from the donor here (Hu_NoV_PT1-9, -11, and -12), was created using the neighbor-joining method with 100 replicates based on a multiple sequence alignment of amino acids. Numbers on the tree correspond to consensus support values (%). The tree is rooted to a human GII.5 norovirus, which is 63% identical in sequence to the other viruses in the tree. All other viruses are ∼93% identical in sequence to each other. Consensus sequences GII.4 MC4 and MC12 were designed for this study.

**FIG 2:**
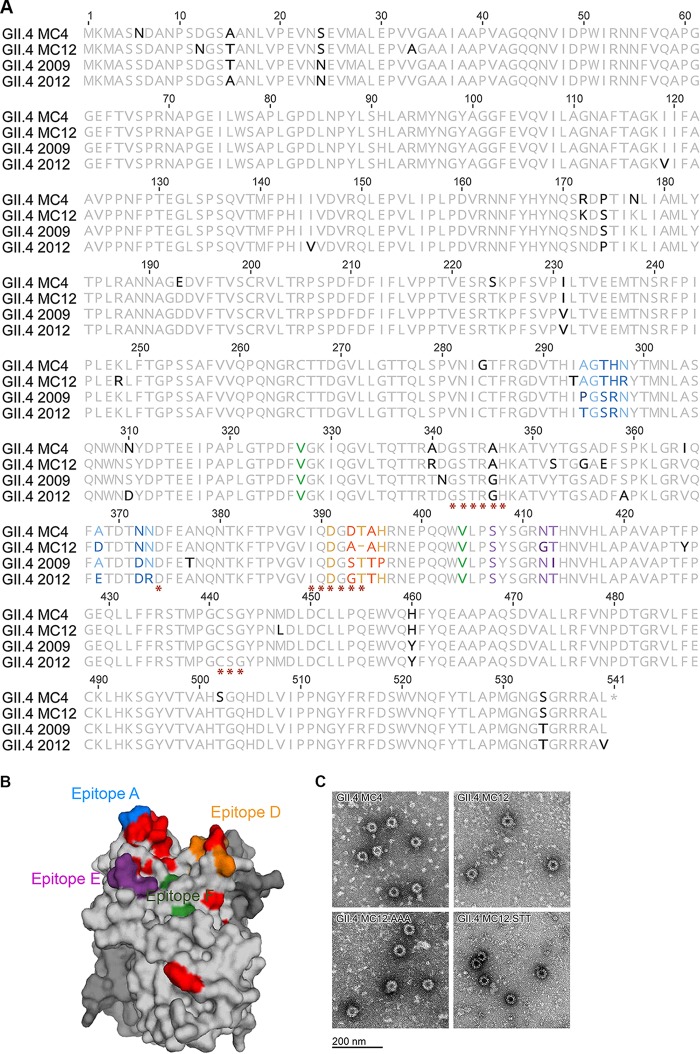
GII.4 MC4 and MC12 capsid protein. (A) Capsid protein amino acid sequences of GII.4 MC4 and MC12 compared to GII.4 2009 New Orleans and 2012 Sydney. Residues known to interact with carbohydrate ligand (*) and antibodies that block VLP-ligand interaction (epitopes A [blue], D [orange], E [purple], and F [green]) are indicated. Residues that vary between the compared sequences are in boldface. (B) Side view of GII.4 MC12 P domain dimer homology model based on GII.4 2012 with one chain color-coded by epitopes as in panel A. The carbohydrate binding domain is not visible in this orientation. Residues that vary in MC12 compared to the consensus sequence of GII.4 2009 and 2012 are colored red. (C) Electron micrograph of VLP created for this study.

We identified a patient with common variable immunodeficiency (CVID) and a complicated medical history being seen in the Infectious Diseases Clinic for chronic diarrhea with persistent norovirus detected in stool. A remnant clinical stool specimen known to be positive for GII norovirus RNA was obtained from the Clinical Microbiology Laboratory. Norovirus capsid gene was amplified by reverse transcription-PCR, the amplicons subcloned into Topo XL, and 11 subclones were sequenced from the single specimen. The goal of this study was to identify unique capsid amino acid combinations that may influence antibody and cellular ligand binding. Our previous studies have shown that *in vivo* evolved norovirus strains may have unique residue substitutions that can enable amino acid level mapping of antibody binding sites (15, 16). The 11 capsid genes formed two clusters within the GII.4 genotype (Fig. 1). Two virus-like particles (VLPs) were designed for study of antibody and ligand binding: GII.4 MC4 representing the consensus sequence of patient strains 1 to 9 and GII.4 MC12 representing the consensus sequence of patient strains 11 and 12. Based on predicted amino acid alignment of capsid, GII.4 MC4 and MC12 exhibit 95.2% identity to each other and cluster closely with the two recent GII.4 pandemic strains GII.4 2009 New Orleans and GII.4 2012 Sydney (94 to 98% identity) (Fig. 1). An ancestral strain could not be determined from capsid protein analysis. Within the capsid protein, 25 residues differ between GII.4 MC4 and MC12. Thirteen changes are within the hypervariable P2 subdomain, including surface-exposed residues of blockade antibody epitopes associated with escape from herd immunity (Fig. 2A). GII.4 MC4 differs from GII.4 2009 and GII.4 2012 at key antigenic sites in epitopes A and D, while GII.4 MC12 differs in epitopes A, D, and E (Fig. 2B), suggesting that the GII.4 MC strains are antigenically distinct from each other and previously circulating GII.4 strains. Of note, GII.4 MC12 has a deletion at residue 394 in epitope D, reminiscent of GII.4 strains that circulated prior to the GII.4 2002 Farmington Hills pandemic stains. Variation in epitope D is associated with both changes in antigenicity and cellular ligand binding (9, 13, 14, 17).

**FIG 3:**
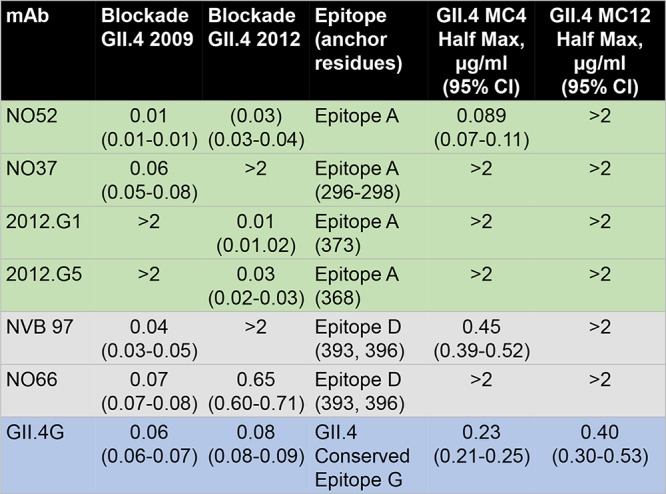
GII.4 MC4 and MC12 are antigenically distinct. GII.4 MC4 and MC12 VLP were screened for reactivity to MAbs to epitopes A (green shading), D (gray shading), and G (blue shading), and half-maximum binding titers and 95% CI values were calculated. The MAb reactivities to GII.4 2009 and GII.4 2012 are reported both here and elsewhere (16, 19, 24, 25, 27).

To characterize the effect of capsid sequence changes, we developed a VLP for each strain (Fig. 2C) and determined the antibody and carbohydrate binding profiles of each. GII.4 MC4 and MC12 are antigenically divergent from GII.4 2009 and GII.4 2012 and each other, based on the reactivity of monoclonal antibodies (MAbs) against evolving blockade antibody epitopes in GII.4 2009 and 2012 (Fig. 3), as indicated by the sequence changes in Fig. 2A. GII.4 MC4 binds to NO52 but not to NO37, two MAbs with different binding footprints on GII.4 2009 epitope A, and to NVB 97 but not NO66, two MAbs to epitope D with distinct anchoring residues (17, 18). GII.4 MC12 did not bind to any of the GII.4 2009 or 2012 epitope A or D MAbs. Both GII.4 MC VLPs bind GII.4G, an MAb to a conserved GII.4 blockade antibody epitope, indicating that the loss of antibody binding to other epitopes is the result of sequence variation between strains and not the lack of particle integrity.

**FIG 4:**
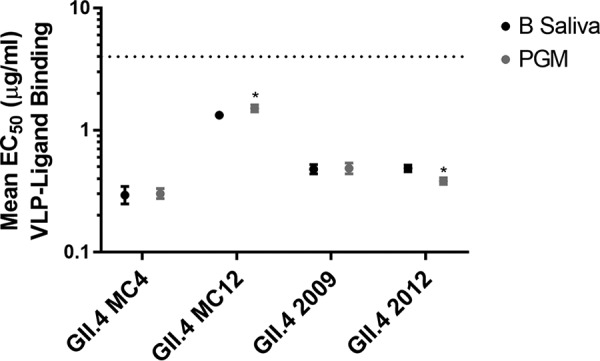
Carbohydrate binding profiles of GII.4 MC4 and MC12. GII.4 MC4 and MC12 bind pig gastric mucin III and human type B saliva. The dashed line is the limit of detection. Markers denote the mean, and error bars indicate the standard errors of the mean (SEM) based on eight replicates from four independent experiments. *, PGM binding significantly different from B saliva binding.

The carbohydrate binding pocket where the α-1,2-fucose of H antigen binds is conserved across GII.4 strains. Affinity for non-H antigen HBGAs is influenced by epitope D, specifically residues 393 and 395 (14, 19), both of which vary in GII.4 MC4 and MC12 compared to GII.4 2009 and GII.4 2012. To compare the relative binding of GII.4 VLPs to natural HBGAs, we screened VLPs for binding to PGM (H, A, and Lewis antigens [20]) and type B human saliva (B antigen), thus covering the major known binding moieties of GII.4 strains. Both GII.4 MC4 and MC12 VLPs bind to PGM and human type B saliva (Fig. 4). Neither GII.4 MC4 nor MC12 bound to secretor-negative saliva from a blood type A donor (data not shown). Compared to GII.4 MC4, MC12 required approximately 5 times more VLP to reach half-maximal binding to the natural carbohydrates.

**FIG 5:**
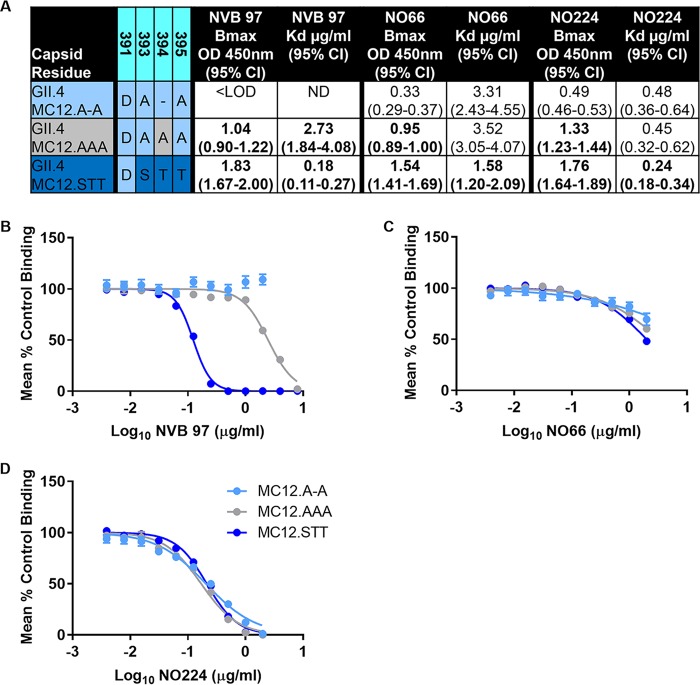
The number and identity of epitope D residues impacts VLP interaction with blockade antibodies by multiple mechanisms. (A) GII.4 MC12, MC12.AAA, and MC12.STT binding to MAbs NVB 97, NO66, and NO224 was analyzed by single-site binding curve fitting, and maximum binding (B_max_) and dissociation constants (*K_d_*) were calculated to differentiate the effects of sequence changes on antibody-epitope access and binding strength, respectively. Boldface numbers are significantly different from GII.4 MC12.A-A. (B to D) Antibody function, as measured by blockade of ligand binding potency, the IC_50_ titer, was determined by dose-response nonlinear curve fit analysis of the mean percent control binding with 95% CI values (error bars).

Reduced ligand relative affinity coupled with lack of binding to epitope D MAbs indicates the unique sequence in residues 393 to 395 of GII.4 MC12 may impact the virus-host interaction at multiple levels. To test the effects of epitope D variation, we first created the smallest full D loop by replacing the deletion at 394 in MC12 (for comparison, this is subsequently referred to as VLP GII.4 MC12.A-A) with an alanine (VLP GII.4 MC12.AAA) and, second, we reconstructed the contemporary strain epitope D consensus sequence 393S, 394T, and 395T (VLP GII.4 MC12.STT) (Fig. 5). Insertion of either an alanine at 394 or STT improved the maximum binding of NVB 97, NO66, and N0224 but only modestly (∼2-fold) improved the *K_d_* for NO66 and NO224 binding. However, compared to MC12.AAA, the *K_d_* for NVB 97 binding to GII.4 MC12.STT improved 15-fold, supporting previous reports of NVB 97 anchoring at residue S393 (21). STT insertion in GII.4.MC12.STT was required to restore antibody blockade of ligand binding potency for epitope D MAb NVB 97 but was insufficient to completely restore or improve the blockade of NO66 or NO224 (Fig. 5B).

**FIG 6:**
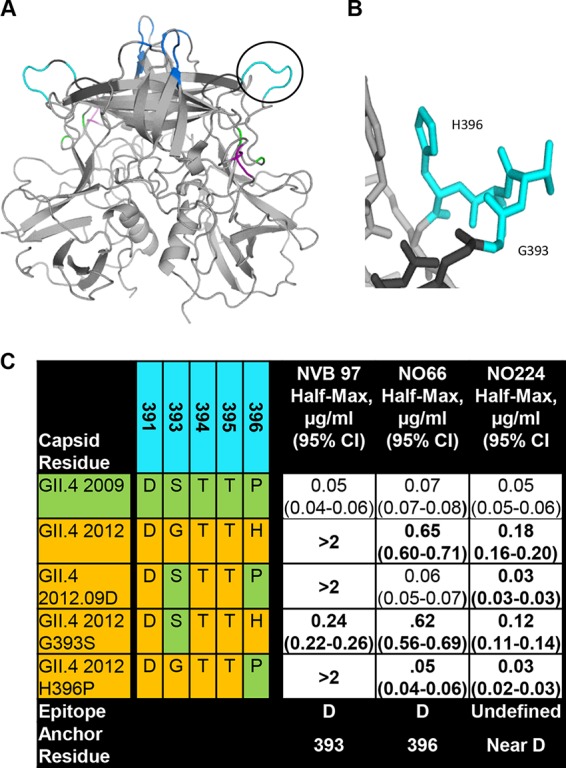
Blockade antibodies anchor at residues 393 and 396 of the protruding loop of epitope D. (A) GII.4 MC12 P domain dimer model with epitope D (cyan) circled and other features color coded as in Fig. 2B. (B) Epitope D enlarged with residues 393 and 396 identified. (C) Half-maximal binding titers with 95% confidence intervals (95% CI) were determined for MAbs NVB 97, NO66, and NO224 to VLPs with substitutions reflective of natural variation between pandemic strains GII.4 2009 and 2012 within epitope D at residues 393 and 396. Boldface numbers are significantly different from GII.4 2009.

Residues 393 and 396 mark distal boundaries of the loop of epitope D that extends from the perimeter of the carbohydrate binding pocket (Fig. 6A and B). These residues vary between pandemic strains GII.4 2009 and 2012 and correspond to loss of blockade potency for NVB 97 and NO66 (22). Therefore, GII.4 2012 VLPs with GII.4 2009 epitope D substitution mutations were developed to dissect the role of residues 393 and 396 in binding of epitope D antibodies. GII.4.2012.09D (residues 393 to 396) and GII.4 2012.H396P improved the 50% effective concentration for NO66 binding more than 10-fold compared to GII.4 2012, identifying residue 396 as an anchor for NO66 and extending epitope D to include the entire loop structure from 393 to 396 (Fig. 6C). GII.4 2012.G393S restored NVB 97 binding but GII.4.2012.09D and GII.4 2012.H396P did not, indicating that the P396 may negatively impact NVB 97 binding to epitope D. NO224 binding was modestly improved by G393S and H396P, indicating that NO224 likely binds near epitope D and that the loop structure of epitope D may affect NO224-epitope binding.

**FIG 7:**
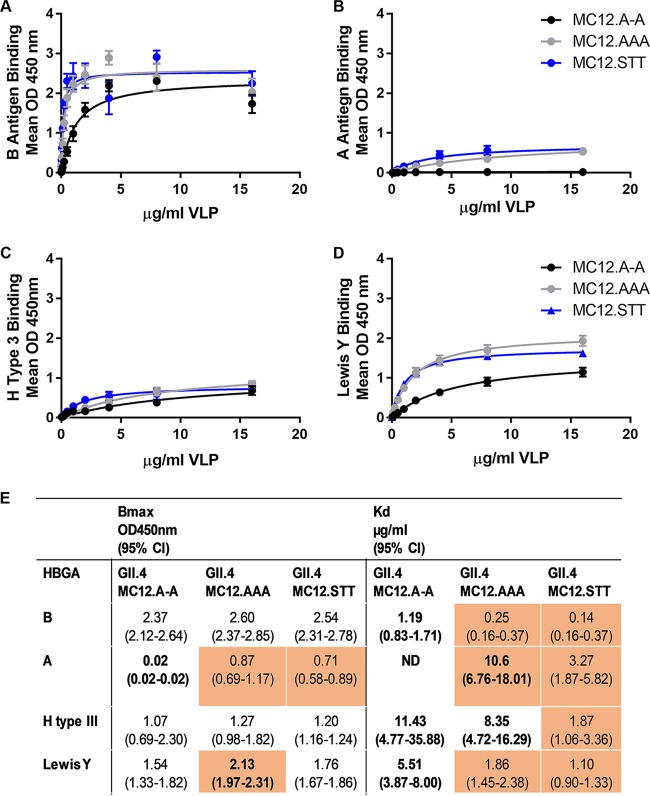
Residue number and sequence within epitope D modulates ligand binding. GII.4 MC12.A-A, MC12.AAA, and MC12.STT binding to synthetic B (A), A (B), H type III (C), and Lewis Y (D) was analyzed by single-site binding curve fitting, and the B_max_ and *K_d_* values were determined (E). Markers denote the mean, and error bars indicate the SEM (minimum of four replicates from two independent experiments). Orange shading indicates significantly different from GII.4 MC12. Boldface values denote significantly different from GII.4.MC12.STT. ND, not determined.

Next, we evaluated GII.4 MC12.A-A, MC12.AAA, and MC12.STT for binding to synthetic biotinylated HBGAs representing the major classes of human norovirus binding ligands (A, B, H, and Lewis) to determine the effect of epitope D sequence variation on ligand binding. GII.4 MC12 bound to B antigen, H type III, and Lewis Y, but not to A antigen. (Fig. 7A to D). Expanding the loop of epitope D improves the relative affinity (*K_d_*) for all ligands tested. Substituting STT in the D loop had the most impact on *K_d_*, improving the relative affinity for ligand binding 5- to 10-fold compared to GII.4 MC12.A-A. GII.4.MC12.AAA improves the *K_d_* less robustly (1.4- to 4.8-fold). Changes in relative affinity were largely uncoupled from changes in maximum binding (<1.5-fold difference), except for A antigen (Fig. 7E). In this instance, a three-residue D loop appears necessary to stabilize A antigen binding. The number of residues in the D loop may be more important than the residue identities since both MC12.AAA and MC12.STT similarly conferred maximum A antigen binding. These data illustrate how sequence variation in epitope D regulates binding to different biological ligands independent of the conserved primary ligand binding site, which remained unchanged between the epitope D mutant VLPs.

## DISCUSSION

Receptor binding sites are conserved targets for neutralizing antibodies within hypervariable RNA viruses (19, 23–25). To avoid immune detection while preserving ligand binding functions, these sites are frequently masked by glycosylation or particle conformation (15, 23, 24). Posttranslational modification of norovirus capsid protein has not been observed (26), but ligand access to the primary ligand binding site may be conformation dependent (27). The secondary ligand binding site, epitope D, is surface exposed, is variable in sequence, and does not exhibit conformation-dependent binding of known antibodies (17, 27), although structural studies are needed for confirmation. Here, utilizing unique GII.4 strains and well-described monoclonal antibodies, we show how the balance between epitope D sequence diversity needed to avoid immune detection and sequence conservation to maintain cellular ligand binding is achieved by first expanding epitope D to include the entire extended loop structure from residues 393 to 396. Binding of epitope D antibodies progressively increases between epitope D mutants GII.4 MC12.A-A, AAA, and STT. Simply expanding the loop structure with the insertion of an alanine residue (GII.4 MC12.AAA) improves antibody relative affinity. However, the shallow antibody binding curves (poor *K_d_*) correlate with insufficient binding to block ligand interaction. Expansion of the loop of epitope D also improves the VLP relative affinity for carbohydrate ligands. Maximum binding is consistent between GII.4 MC12 epitope D mutant VLPs for B and H type III, emphasizing the role of the conserved α-1,2-fucose binding pocket in determining the total number of carbohydrate molecules able to bind to the particle (14, 19). The *K_d_* for B and Lewis antigen binding GII.4 MC12.AAA and STT are similar, indicating that expansion of the epitope D loop to include another amino acid may be more important than the identity of the expanding residue for ligand interactions. In contrast, sequence variation in the epitope D loop did affect stabilization of the interaction between epitope D mutant VLPs and A antigen, indicating that weaker carbohydrate interactions may be more susceptible to sequence variation.

Together, these data support a model of viral evolution within epitope D that tolerates sequence variation that may selectively enhance or preserve binding of specific carbohydrate moieties while concurrently allowing for escape from neutralizing antibody that is less able to tolerate sequence variation without losing the ability to block VLP-ligand binding. The ability to fine-tune ligand usage could be particularly advantageous as herd immunity to a strain is established. Evolution that abrogates recognition by epitope D antibodies and subsequently opens new population segments for infection would contribute to prolonging strain circulation time before herd immunity to epitope A and other sites drives a strain to extinction and a new variant emerges.

Immunosuppressed patients have been postulated as potential reservoirs of emergent GII.4 strains, although hospital strains typically correlate with community strains (6, 16, 28, 29). Although the numbers are small, previous studies of norovirus diversity in CVID patients identified novel strains within patients but no significant difference in diversification rates by an immunosuppression mechanism (5, 30). The donor in this study represents a particularly interesting though unusual scenario in that persistent norovirus infection and shedding was made possible due to the immunocompromising condition; however, the treatment (immunoglobulin replacement therapy) is essentially passive transfer of immunity from healthy individuals. Because immunoglobulin for therapeutic use may be pooled from many regional donors, the specificities represented in each treatment are a sample of herd immunity in the general population. Thus, in contrast to most other immunocompromising conditions leading to chronic norovirus infection, CVID treated with replacement immunoglobulin may be a relevant *in vivo* human model for how norovirus strains escape herd immunity. Further studies may include treatment with multiple lots of intravenous immunoglobulin to evaluate whether increased antibody breadth more effectively halts emergence of viral escape variants. Alternatively, the development of therapeutic MAb cocktails targeting conserved epitopes (15) may provide more comprehensive protection from emergent strains.

Long-term, *in vivo* evolution can result in virus with unique characteristics (15, 16, 31). Residue substitutions in epitope A correlate with loss of antibody potency and strain emergence. Here, GII.4 MC4 and MC12 had low or no reactivity to GII.4 2009 or 2012 epitope A MAbs with different anchoring amino acids, indicating that viral evolution in this donor continued to select for strains with novel combinations of epitope A residues. Whether these viruses pose a risk to the community is unclear, since fitness in an immunocompetent population has not been studied. Models of disease spread do not support single source strain introduction, as would be predicted from a variant originating from an individual (32). Further, at early days postvaccination, a blockade antibody response following norovirus vaccination was effective at blocking a novel GII.4 variant isolated from an immunosuppressed individual with long-term shedding (33, 34). However, antigenic cartography of sera from immunized mice, indicative of antibody responses in norovirus-naive young children, illustrates similar levels of antigenic drift between strains isolated from an immunocompromised patient infected for about 1 year, and the amount of drift between the GII.4 2009 and the GII.4 2012 pandemic strains (16) support, based on antigenic differences, the potential of an ICH *in vivo*-evolved strain to seed disease in children. Follow-up study of virus shedding in contacts of norovirus-positive immunocompromised patients is needed to assess the infectiousness of these novel viral variants in the healthy population and to determine the risk these strains pose. The unique environment posed by incomplete immunosuppression in many ICH may encourage the greatest breadth of circulating viral variants containing novel mutations (29). These *in vivo*-evolved variants are a rich source of replication-competent viral mutants for studying the role of capsid amino acids in driving viral characteristics relevant to pandemic surveillance and vaccine design.

## MATERIALS AND METHODS

### Study subject.

All studies were approved by the University of North Carolina Institutional Review Board (IRB protocol 14-3055). Written informed consent was obtained prior to participation. Excess clinical stool specimen was obtained from the University of North Carolina Hospitals Clinical Microbiology Laboratory and transported to our research lab on ice and stored at 4^○^C until further use. The donor had a diagnosis of CVID that was complicated by chronic intermittent diarrhea and histopathologic findings, such as duodenal villous flattening and lymphocytic colitis, consistent with CVID-associated enteropathy (CVID-AE), which were present 6 years prior to this study. This condition had been managed with different immunosuppressive therapies. An infectious etiology for diarrhea was first diagnosed 3 years prior (Clostridium difficile). Norovirus GII was first detected later that same year, and symptoms were unaffected by administration of nitazoxanide and improved after oral immunoglobulin. The patient was asymptomatic off prednisone for CVID-AE for much of the following 1 to 2 years. Diarrheal symptoms recurred late in the second symptom-free year with an additional norovirus GII+ stool test, and both persisted until the end of that year when the clinical specimen was obtained for research. Molecular or stool culture testing for an extensive panel of viral, bacterial, and parasitic gastrointestinal pathogens was consistently negative throughout this time. Multiple HIV tests were negative. Previous serologic testing for autoimmune causes was negative. Gluten-free diet did not improve symptoms. At the time the specimen was obtained for these studies, pertinent medications included valacyclovir, ribavirin, prednisone, tacrolimus, and subcutaneous immunoglobulin G replacement. No additional samples or patient follow-up studies are available. Provided material was sufficient to sequences capsid genes only.

### Virus-like particle production.

The stool sample was diluted to approximately 10% with phosphate-buffered saline. This suspension was vortexed and then centrifuged at 3,000 × *g* for 10 min. RNA was extracted from 250 μl of the clarified supernatant using 750 μl of TRIzol LS (Life Technologies), as recommended. RNA was then extracted using a QIAamp Viral RNA Mini kit (Qiagen). cDNA was made using 10 μl of RNA and a Superscript II RT kit (Invitrogen) as recommended. The cDNA was digested with 0.01 μg of RNase (DNase free; Roche) and then purified with a QIAquick PCR purification kit (Qiagen) and amplified by PCR using primers targeting the 5-prime (VEE-NV5ʹ, AGTCTAGTCCGCCAAGATGAAGATGGCGTCGAATGAC) and 3-prime (NV3ʹAscI, NNNNNNGGCGCGCCTTATAATGCACGTCTACGCCC) ends of ORF2. The amplicons were cloned into Tope XL (Invitrogen), and 12 colonies were selected and sequenced. GII.4 MC4 represents the consensus sequence of the 12 clones, and MC12 represents the strain with the most divergent sequence. Capsid genes were synthesized by Bio-Basic, Inc. (Amherst, NY). VLPs were expressed in baby hamster kidney cells (ATCC CCL-10) from Venezuelan equine encephalitis virus replicons expressing norovirus open reading frame 2 (NoV ORF2), as described previously (17, 35, 36). Particle integrity was confirmed by electron microscopy visualization of negative-stained particles of ∼40 nm.

### Phylogenetic tree and alignment.

The tree was constructed in Geneious R11 using the neighbor-joining method (Jukes-Cantor genetic distance) with 100 replicates based on a multiple alignment of the indicated norovirus capsid sequences. Numbers in parentheses following virus species names indicate the number of sequences represented at that tree position. The radial phylogram was visualized and rendered for publication using CLC Sequence Viewer 7 and Adobe Illustrator CC 2017.

### Homology modeling.

Homology models representing the capsid P domain for patient isolates GII.4 MC4 and GII.4 MC12 were generated using Swiss-Model. To do this, capsid amino acid sequences for these viruses were uploaded into the Swiss-Model modeling server (https://swissmodel.expasy.org/interactive), and potential templates were chosen by clicking “search for templates.” The template with the highest homology score was chosen (PDB accession number 4OP7), which is the crystal structure for NSW0514, a GII.4 2012 Sydney sequence. Models of the capsid dimers were created and rendered in MacPyMOL version 1.8.0.4 (https://www.pymol.org) using the 4OP7 template.

### VLP-ligand binding assays.

VLPs bound to pig gastric mucin type III (PGM; Sigma-Aldrich), human type B saliva, or synthetic biotinylated HBGAs (Glycotech) were detected by rabbit polyclonal antiserum (Cocalico Biologicals, Stevens, PA), as described previously (21, 27). The optical density, as a measure of binding, should be compared between VLP for the same synthetic biotinylated carbohydrate, not between different carbohydrates, to account for variability between lots of commercial reagents.

### EIA and blockade of VLP-ligand binding assays.

Enzyme immunoassay (EIA) and blockade antibody assays were performed at 37°C with 0.25 μg/ml VLP (27, 37). An OD at 450 nm of >3-fold background after background subtraction was scored as positive by the EIA. For blockade assays, VLPs were pretreated with decreasing concentrations of MAb for 1 h and transferred to PGM-coated plates for 1 h. The percent control binding was compared to a no-antibody pretreatment. Mean 50% inhibitory concentration (IC_50_) titers and 95% confidence intervals (95% CI) were determined from dose-response sigmoidal curve fits using GraphPad 7.02 (33, 34). Antibodies below the limit of detection (half-maximum binding at 8 μg/ml) or that did not block at least 50% of VLP binding to PGM at the lowest dilution tested were assigned a titer equal to 2× (EIA) or 0.5× (blockade assay) the limit of detection for statistical analysis. Maximum binding and *K_d_* were determined by single-site binding curve analysis using GraphPad Prism 7.02 (15).

### Statistical analysis.

Statistical analyses were performed using GraphPad Prism 7.02 (17, 33). Antibody and synthetic carbohydrate maximum binding and *K_d_* titers were determined by single-site binding curve analysis. IC_50_ values were log transformed for analysis. Antibody titer and binding measurements were compared by unpaired *t* test with Welch’s correction (*t* test) or ordinary one-way analysis of variance with Dunnett’s multiple-comparison test. A difference was considered significant if the *P* value was <0.05.

### Accession number(s).

GII.4 MC4 (MH746820) and GII.4 MC12 (MH746821) were examined in this study.

## References

[1] Ahmed SM, Hall AJ, Robinson AE, Verhoef L, Premkumar P, Parashar UD, Koopmans M, Lopman BA 2014 Global prevalence of norovirus in cases of gastroenteritis: a systematic review and meta-analysis. Lancet Infect Dis 14:725–730. doi:10.1016/S1473-3099(14)70767-4. 24981041 PMC8006533

[2] Lopman BA, Steele D, Kirkwood CD, Parashar UD 2016 The vast and varied global burden of norovirus: prospects for prevention and control. PLoS Med 13:e1001999. doi:10.1371/journal.pmed.1001999. 27115709 PMC4846155

[3] Becker-Dreps S, Bucardo F, Vilchez S, Zambrana LE, Liu L, Weber DJ, Pena R, Barclay L, Vinje J, Hudgens MG, Nordgren J, Svensson L, Morgan DR, Espinoza F, Paniagua M 2014 Etiology of childhood diarrhea after rotavirus vaccine introduction: a prospective, population-based study in Nicaragua. Pediatr Infect Dis J 33:1156–1163. doi:10.1097/INF.0000000000000427. 24879131 PMC4216626

[4] McAtee CL, Webman R, Gilman RH, Mejia C, Bern C, Apaza S, Espetia S, Pajuelo M, Saito M, Challappa R, Soria R, Ribera JP, Lozano D, Torrico F 2016 Burden of norovirus and rotavirus in children after rotavirus vaccine introduction, Cochabamba, Bolivia. Am J Trop Med Hyg 94:212–217. doi:10.4269/ajtmh.15-0203. 26598569 PMC4710432

[5] Bok K, Green KY 2012 Norovirus gastroenteritis in immunocompromised patients. N Engl J Med 367:2126–2132. doi:10.1056/NEJMra1207742. 23190223 PMC4944753

[6] Doerflinger SY, Weichert S, Koromyslova A, Chan M, Schwerk C, Adam R, Jennewein S, Hansman GS, Schroten H 2017 Human norovirus evolution in a chronically infected host. mSphere 2:e00352-16. 10.1128/mSphere.00352-16 PMC5371696 28405629

[7] Schorn R, Hohne M, Meerbach A, Bossart W, Wuthrich RP, Schreier E, Muller NJ, Fehr T 2010 Chronic norovirus infection after kidney transplantation: molecular evidence for immune-driven viral evolution. Clin Infect Dis 51:307–314. doi:10.1086/653939. 20575662

[8] van Beek J, van der Eijk AA, Fraaij PL, Caliskan K, Cransberg K, Dalinghaus M, Hoek RA, Metselaar HJ, Roodnat J, Vennema H, Koopmans MP 2017 Chronic norovirus infection among solid organ recipients in a tertiary care hospital, the Netherlands, 2006-2014. Clin Microbiol Infect 23:265–269. doi:10.1016/j.cmi.2016.12.010. 28003123

[9] Lindesmith LC, Donaldson EF, Lobue AD, Cannon JL, Zheng DP, Vinje J, Baric RS 2008 Mechanisms of GII.4 norovirus persistence in human populations. PLoS Med 5:e31. doi:10.1371/journal.pmed.0050031. 18271619 PMC2235898

[10] Siebenga JJ, Vennema H, Renckens B, de Bruin E, van der Veer B, Siezen RJ, Koopmans M 2007 Epochal evolution of GGII.4 norovirus capsid proteins from 1995 to 2006. J Virol 81:9932–9941. doi:10.1128/JVI.00674-07. 17609280 PMC2045401

[11] Le Pendu J, Ruvoen-Clouet N, Kindberg E, Svensson L 2006 Mendelian resistance to human norovirus infections. Semin Immunol 18:375–386. doi:10.1016/j.smim.2006.07.009. 16973373 PMC7129677

[12] Tan M, Jiang X 2005 Norovirus and its histo-blood group antigen receptors: an answer to a historical puzzle. Trends Microbiol 13:285–293. doi:10.1016/j.tim.2005.04.004. 15936661

[13] Shanker S, Choi JM, Sankaran B, Atmar RL, Estes MK, Prasad BV 2011 Structural analysis of HBGA binding specificity in a norovirus GII.4 epidemic variant: implications for epochal evolution. J Virol 85:8635–8645. doi:10.1128/JVI.00848-11. 21715503 PMC3165782

[14] de Rougemont A, Ruvoen-Clouet N, Simon B, Estienney M, Elie-Caille C, Aho S, Pothier P, Le Pendu J, Boireau W, Belliot G 2011 Qualitative and quantitative analysis of the binding of GII.4 norovirus variants onto human blood group antigens. J Virol 85:4057–4070. doi:10.1128/JVI.02077-10. 21345963 PMC3126233

[15] Lindesmith LC, Mallory ML, Debbink K, Donaldson EF, Brewer-Jensen PD, Swann EW, Sheahan TP, Graham RL, Beltramello M, Corti D, Lanzavecchia A, Baric RS 2018 Conformational occlusion of blockade antibody epitopes, a novel mechanism of GII.4 human norovirus immune evasion. mSphere 3:e00518-17. doi:10.1128/mSphere.00518-17. 29435493 PMC5806210

[16] Debbink K, Lindesmith LC, Ferris MT, Swanstrom J, Beltramello M, Corti D, Lanzavecchia A, Baric RS 2014 Within-host evolution results in antigenically distinct GII.4 noroviruses. J Virol 88:7244–7255. doi:10.1128/JVI.00203-14. 24648459 PMC4054459

[17] Lindesmith LC, Beltramello M, Donaldson EF, Corti D, Swanstrom J, Debbink K, Lanzavecchia A, Baric RS 2012 Immunogenetic mechanisms driving norovirus GII.4 antigenic variation. PLoS Pathog 8:e1002705. doi:10.1371/journal.ppat.1002705. 22615565 PMC3355092

[18] Lindesmith LC, Costantini V, Swanstrom J, Debbink K, Donaldson EF, Vinje J, Baric RS 2013 Emergence of a norovirus GII.4 strain correlates with changes in evolving blockade epitopes. J Virol 87:2803–2813. doi:10.1128/JVI.03106-12. 23269783 PMC3571402

[19] Shanker S, Czako R, Sapparapu G, Alvarado G, Viskovska M, Sankaran B, Atmar RL, Crowe JE Jr, Estes MK, Prasad BV 2016 Structural basis for norovirus neutralization by an HBGA blocking human IgA antibody. Proc Natl Acad Sci U S A 113:E5830–E5837. doi:10.1073/pnas.1609990113. 27647885 PMC5056091

[20] Lindesmith LC, Debbink K, Swanstrom J, Vinje J, Costantini V, Baric RS, Donaldson EF 2012 Monoclonal antibody-based antigenic mapping of norovirus GII.4-2002. J Virol 86:873–883. doi:10.1128/JVI.06200-11. 22090098 PMC3255811

[21] Lindesmith LC, Brewer-Jensen PD, Mallory ML, Debbink K, Swann EW, Vinjé J, Baric RS 2018 Antigenic characterization of a novel recombinant GII.P16-GII.4 Sydney norovirus strain with minor sequence variation leading to antibody escape. J Infect Dis 217:1145–1152. doi:10.1093/infdis/jix651. 29281104 PMC5939617

[22] Debbink K, Lindesmith LC, Donaldson EF, Costantini V, Beltramello M, Corti D, Swanstrom J, Lanzavecchia A, Vinje J, Baric RS 2013 Emergence of new pandemic GII.4 Sydney norovirus strain correlates with escape from herd immunity. J Infect Dis 208:1877–1887. doi:10.1093/infdis/jit370. 23908476 PMC3814837

[23] Kwong PD, Mascola JR 2018 HIV-1 vaccines based on antibody identification, B cell ontogeny, and epitope structure. Immunity 48:855–871. doi:10.1016/j.immuni.2018.04.029. 29768174

[24] Wu NC, Wilson IA 2017 A perspective on the structural and functional constraints for immune evasion: insights from influenza virus. J Mol Biol 429:2694–2709. doi:10.1016/j.jmb.2017.06.015. 28648617 PMC5573227

[25] Edwards VC, Tarr AW, Urbanowicz RA, Ball JK 2012 The role of neutralizing antibodies in hepatitis C virus infection. J Gen Virol 93:1–19. doi:10.1099/vir.0.035956-0. 22049091

[26] Baric RS, Yount B, Lindesmith L, Harrington PR, Greene SR, Tseng FC, Davis N, Johnston RE, Klapper DG, Moe CL 2002 Expression and self-assembly of Norwalk virus capsid protein from Venezuelan equine encephalitis virus replicons. J Virol 76:3023–3030. doi:10.1128/JVI.76.6.3023-3030.2002. 11861868 PMC135954

[27] Lindesmith LC, Donaldson EF, Beltramello M, Pintus S, Corti D, Swanstrom J, Debbink K, Jones TA, Lanzavecchia A, Baric RS 2014 Particle conformation regulates antibody access to a conserved GII.4 norovirus blockade epitope. J Virol 88:8826–8842. doi:10.1128/JVI.01192-14. 24872579 PMC4136251

[28] Siebenga JJ, Beersma MF, Vennema H, van Biezen P, Hartwig NJ, Koopmans M 2008 High prevalence of prolonged norovirus shedding and illness among hospitalized patients: a model for *in vivo* molecular evolution. J Infect Dis 198:994–1001. doi:10.1086/591627. 18774885

[29] Karst SM, Baric RS 2015 What is the reservoir of emergent human norovirus strains? J Virol 89:5756–5759. doi:10.1128/JVI.03063-14. 25787285 PMC4442419

[30] van Beek J, de Graaf M, Smits S, Schapendonk CME, Verjans G, Vennema H, van der Eijk AA, Phan MVT, Cotten M, Koopmans M 2017 Whole-genome next-generation sequencing to study within-host evolution of norovirus (NoV) among immunocompromised patients with chronic NoV infection. J Infect Dis 216:1513–1524. doi:10.1093/infdis/jix520. 29029115

[31] Mai H, Gao Y, Cong X, Wang H, Liu N, Huang X, Xu L, Chen Y, Wei L 2016 GII.4 Sydney_2012 norovirus infection in immunocompromised patients in Beijing and its rapid evolution *in vivo*. J Med Virol 88:224–233. doi:10.1002/jmv.24332. 26185038

[32] Eden JS, Bull RA, Tu E, McIver CJ, Lyon MJ, Marshall JA, Smith DW, Musto J, Rawlinson WD, White PA 2010 Norovirus GII.4 variant 2006b caused epidemics of acute gastroenteritis in Australia during 2007 and 2008. J Clin Virol 49:265–271. doi:10.1016/j.jcv.2010.09.001. 20888289

[33] Lindesmith LC, Ferris MT, Mullan CW, Ferreira J, Debbink K, Swanstrom J, Richardson C, Goodwin RR, Baehner F, Mendelman PM, Bargatze RF, Baric RS 2015 Broad blockade antibody responses in human volunteers after immunization with a multivalent norovirus VLP candidate vaccine: immunological analyses from a phase I clinical trial. PLoS Med 12:e1001807. doi:10.1371/journal.pmed.1001807. 25803642 PMC4371888

[34] Lindesmith LC, Mallory ML, Jones TA, Richardson C, Goodwin RR, Baehner F, Mendelman PM, Bargatze RF, Baric RS 2017 Impact of pre-exposure history and host genetics on antibody avidity following norovirus vaccination. J Infect Dis 215:984–991. doi:10.1093/infdis/jix045. 28453838 PMC5853323

[35] Debbink K, Costantini V, Swanstrom J, Agnihothram S, Vinje J, Baric R, Lindesmith L 2013 Human norovirus detection and production, quantification, and storage of virus-like particles. Curr Protoc Microbiol 31:15K.11.11–15K.11.45. 10.1002/9780471729259.mc15k01s31 PMC3920292 24510290

[36] Agnihothram S, Menachery VD, Yount BL Jr, Lindesmith LC, Scobey T, Whitmore A, Schafer A, Heise MT, Baric RS 2018 Development of a broadly accessible venezuelan equine encephalitis virus replicon particle vaccine platform. J Virol 92:e00027-18. 29540599 10.1128/JVI.00027-18 PMC5952155

[37] Lindesmith LC, Beltramello M, Swanstrom J, Jones TA, Corti D, Lanzavecchia A, Baric RS 2015 Serum immunoglobulin A cross-strain blockade of human noroviruses. Open Forum Infect Dis 2:ofv084. doi:10.1093/ofid/ofv084. 26180833 PMC4498284

